# Role of the *gerP* Operon in Germination and Outgrowth of *Bacillus anthracis* Spores

**DOI:** 10.1371/journal.pone.0009128

**Published:** 2010-02-09

**Authors:** Katherine A. Carr, Brian K. Janes, Philip C. Hanna

**Affiliations:** Department of Microbiology and Immunology, University of Michigan Medical School, Ann Arbor, Michigan, United States of America; Max Planck Institute for Infection Biology, Germany

## Abstract

Germination of *Bacillus anthracis* spores occurs when nutrients such as amino acids or purine nucleosides stimulate specific germinant receptors located in the spore inner membrane. The *gerP_ABCDEF_* operon has been suggested to play a role in facilitating the interaction between germinants and their receptors in spores of *Bacillus subtilis* and *Bacillus cereus*. *B. anthracis* mutants containing deletions in each of the six genes belonging to the orthologue of the *gerP_ABCDEF_* operon, or deletion of the entire operon, were tested for their ability to germinate. Deletion of the entire *gerP* operon resulted in a significant delay in germination in response to nutrient germinants. These spores eventually germinated to levels equivalent to wild-type, suggesting that an additional entry point for nutrient germinants may exist. Deletions of each individual gene resulted in a similar phenotype, with the exception of Δ*gerPF*, which showed no obvious defect. The removal of two additional *gerPF-*like orthologues was necessary to achieve the germination defect observed for the other mutants. Upon physical removal of the spore coat, the mutant lacking the full *gerP* operon no longer exhibited a germination defect, suggesting that the GerP proteins play a role in spore coat permeability. Additionally, each of the *gerP* mutants exhibited a severe defect in calcium-dipicolinic acid (Ca-DPA)–dependent germination, suggesting a role for the GerP proteins in this process. Collectively, these data implicate all GerP proteins in the early stages of spore germination.

## Introduction

The gram positive bacterium *Bacillus anthracis* exists in two morphologically distinct forms, the metabolically active cell, and the dormant spore. Bacterial spores form in response to nutrient depletion as a means of protection until they enter into a hospitable environment [1]. At the center of the spore is the core which contains a high concentration of dipicolinic acid and its associated calcium ions (Ca-DPA) that helps to protect DNA from damage, and takes the place of water inside the spore [2]. This dehydrated state is also essential for spore stability and contributes to the hallmark heat resistance that spores possess [3]. Outside the core lies the cortex, a layer of highly modified peptidoglycan which contributes to the resistance properties of the spore by maintaining a high level of dehydration in the core [4]–[6]. The cortex is surrounded by the proteinaceous spore coat, which provides a protective barrier, and also plays a role in the process of germination [7]–[10]. The coat and cortex are both believed to be permeable to water, while still maintaining the spore's resistance to environmental stresses and various chemicals [5], [6]. *B. anthracis* also has an additional outermost layer, the exosporium, which may contribute to adhesion to host cells and communication with the host environment [9], [11]–[14]. The spore structure enables *B. anthracis* to survive dormant in the environment for years, until it encounters an appropriate mammalian host, germinates, and initiates disease. This process can also be triggered *in vitro* using chemical germinants. Although dormancy can last for extended periods of time, the germination process, once initiated, is very rapid. This may contribute to the abilities of *B. anthracis* to multiply and spread quickly once spores enter a host [15].

The onset of germination is dependent upon appropriate germinant-receptor interactions. Germinants are typically amino acids and purines for *B. anthracis*, although other nutrient and non-nutrient related pathways exist, such as the stimulation of cortex lytic enzymes by Ca-DPA [16]–[20]. Before germination can begin, germinants must somehow pass through the exterior layers of the spore to reach their receptors located in the inner membrane [21]. Next, Ca-DPA is released from the spore core. This efflux allows for water and ions to flow back into the core and begin rehydration [22]. Activated lytic enzymes hydrolyze the cortex peptidoglycan leading to full rehydration [2]. The entire germination process can occur within minutes under optimal conditions.

**Figure 1:**
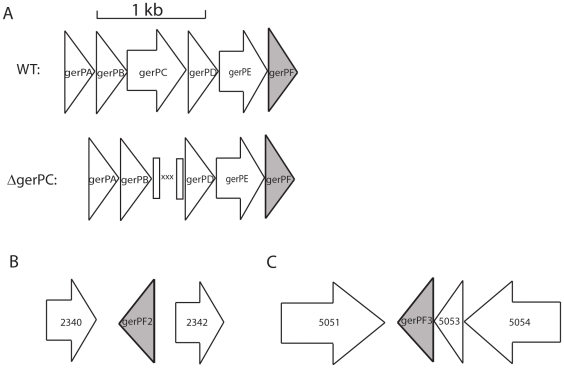
The genes of the *gerP* operon and orthologues. (A) Gene map of the *gerP* operon and example of construction of deletion mutants. Mutants were created by allelic exchange, removing the gene of interest (Δ*gerP*C shown here). For each deletion the first and last 30 nucleotides of the gene of interest were fused together, creating markerless deletions. Non-native sequences are denoted by an “X” above. (B) Gene map of the *gerPF* orthologue *gerPF2*. (C) Gene map of the *gerPF* orthologue *gerPF3*. *gerPF-* family genes are shown in gray.

Although the overall process of germination has been elucidated in a general sense, much remains to be understood, including how germinants are able to access their receptors in the inner membrane. A six-gene operon, termed *gerP_ABCDEF_* (*gerP*), first identified in *Bacillus cereus* and *Bacillus subtilis*, may be involved in this process. The *gerP* operon is under control of a σ^K^ promoter, characteristic of spore coat related genes transcribed in the mother cell during sporulation [23]. GerP proteins encoded within this operon are not predicted to maintain enzymatic activities, and yet *gerP* mutants have severe germination defects in other *Bacillus* species [23]. The *B. anthracis* GerP proteins share an average of 97% amino acid identity to the GerP proteins of *B. cereus* [24], [25] (**Figure 1A**). As *B. cereus* GerP gene products are predicted to play a role in coat structure or formation, and mutation of the operon results in a germination defect, it is possible that the GerP proteins help facilitate the interaction between germinants and their receptors. Here we have created markerless deletions of the *gerP* operon, and each individual gene within the operon, in order to assess the role the GerP proteins play in the rapid germination of *B. anthracis* spores.

## Materials and Methods

### Strains and Culture Conditions

The strains used in this study are listed in **Table 1**. Strains were cultured with brain heart infusion (BHI, Difco) broth or solid media containing 15 g agar per liter. Strains were grown in modified G medium [26] for three days at 37°C with shaking, or for five days at room temperature for complementation, and spores were prepared as previously described [27]. Spores were stored at room temperature in sterile water and titered by hemacytometer (spores/mL) or by colony forming units (cfu/mL), as indicated in the text.

**Table 1:** *Bacillus anthracis* strains used in this study.

Strains	Mutant Name	Relevant Characteristics	Reference
34F_2_	-	Wild-type (pXO1^+^, pXO2^−^)	[38]
KC101	Δ*gerPA*	34F_2_, Δ*gerPA*	This work
KC102	Δ*gerPB*	34F_2_, Δ*gerPB*	This work
KC103	Δ*gerPC*	34F_2_, Δ*gerPC*	This work
KC104	Δ*gerPD*	34F_2_, Δ*gerPD*	This work
KC105	Δ*gerPE*	34F_2_, Δ*gerPE*	This work
KC106	Δ*gerPF*	34F_2_, Δ*gerPF*	This work
KC107	Δ*gerPF_null_*	34F_2_, Δ*gerPF* Δ*gerPF2* Δ*gerPF3*	This work
KC108	Δ*gerP-null*	34F_2_, Δ*gerP_ABCDEF_*	This work

### *gerP* Mutant Construction

Each of the mutant strains used in this work were created using allelic exchange, resulting in markerless deletions (**Figure 1**). Each mutant allele was designed to contain the first 10 codons of the target gene, a short insert sequence of three stop codons and restriction sites for the restriction endonucleases BamHI and SmaI, followed by the final 10 codons of the gene, including the putative stop codon. The constructs used to create each mutant were isolated by PCR (primer sequences available upon request). In addition to the mutant allele described above, each PCR product contained approximately 500 bp of DNA sequence homologous to the upstream and downstream region of the *gerP* gene, flanked by the recognition sequence for the restriction endonuclease NotI. Each PCR product was cloned into the pCR®8/*GW*/*TOPO* vector (Invitrogen) according to manufacturer's instructions. The DNA sequence of each construct was verified to ensure no additional mutations were present due to PCR error. The NotI fragment was then cloned into the allelic exchange vector pBKJ258-kan. This vector was identical to the previously described pBKJ258 [28], with the exception that a kanamycin resistance cassette was exchanged for the original erythromycin cassette. Allelic exchange was performed essentially as described previously [29]. The Δ*gerP-null* mutant was isolated by fusing the first 10 codons of *gerPA* to the last 10 codons of *gerPF*, with stop codons and restriction sites inserted in between, as described above. All mutant alleles were verified using PCR, with primers designed to anneal outside of the sequences used for homologous recombination.

For complementation studies, each gene was constructed under the expression of the native *gerP* promoter using PCR (primers available upon request). Each construct was moved into pBKJ401, a modified version of pBKJ258 containing sequence homology with the *B. anthracis* pXO1 virulence plasmid, and transferred into the mutant of choice via conjugation [29]. Spores were produced by incubating at room temperature for 5 days, as the plasmid carrying the complementary allele was temperature sensitive. Wild-type spores prepared at room temperature showed no difference in germination ability when compared to wild-type spores prepared at 37°C, suggesting that this was a reasonable method of complementation (data not shown).

### Germination Assays

Germination was assayed by measuring the loss of heat resistance of spore suspensions after exposure to germinants. Spores were first heat activated by incubating at 65°C for 20 minutes. 5×10^3^ spores were mixed with 2 mL of germinant, either 0.5 mM L-alanine with 1 mM inosine in phosphate buffered saline (PBS) pH 7.4 (Gibco), or a solution of 60 mM CaCl_2_ and 60 mM dipicolinic acid (pH 8, adjusted with NaOH) in water. Samples were vortexed very briefly and 50 µL were plated on a BHI plate in order to obtain the total number of spores in the sample. Samples were incubated at 37°C for 30 minutes. At 2, 5, 10, 15, 20, and 30 minutes, 200 µL aliquots were removed and heat treated at 65°C for 20 minutes, after which time 100 µL were plated on BHI. Plates were incubated overnight at 37°C and cfu/mL determined.

To assay decrease in optical density, heat activated spores (see above) were added to 400 µL of germinant, giving a starting OD_600_ of 0.3 using a Genesys 10UV spectrophotometer (Spectronic Unicam, Rochester, NY). The reaction was incubated at 37°C while shaking at 200 RPM. At 5 and 30 minutes the OD_600_ of the germinated spore mixture was measured. It has been previously established that a loss of 60–70% of the starting OD_600_ value corresponds with complete germination [16]. In all cases the parental 34F_2_ Sterne strain was used as the positive control.

### Growth Experiments

Bacterial growth was measured in a 96 well plate in a Molecular Devices M2 plate reader. 100 µL of a non heat-activated 2.5×10^8^ spores/mL stock were added to 100 µL 2x BHI. Plates were secured with Parafilm and incubated with shaking for 10 hours, with OD_600_ being measured every 10 minutes.

### Spore Coat Removal

Spore coats were removed using a method adapted from Brown *et al*. [30]. 2.5×10^5^ spores were added to 500 µL UDS buffer (5 mM 2-(*N-*cyclohexylamino) ethanesulfonic acid (CHES) buffer, pH 8.6, containing 8 M urea, 70 mM dithiothreitol, and 1% (wt/vol) sodium dodecyl sulfate (SDS)) and incubated at 37°C for 90 minutes to remove the spore coats. **Treat**ed spores were then washed 5 times with cold sterile water prior to heat activation. Loss of heat resistance was used as a measure of germination (see above). Coat removal was verified by testing sensitivity to lysozyme mediated germination. Germination assays were performed as described above, using 100 µg/mL egg white lysozyme in 0.05 M Tris-HCl, pH 8 and 5 mM EDTA as a germinant. Coat-depleted spores exhibited the expected enhanced sensitivity to lysozyme (ranging from 40–70%), when compared to non-stripped spores (about 10%) (data not shown). Although full coat removal may not have been obtained, it was sufficient to test whether the germination defect could be alleviated with this technique.

## Results

### Germination Phenotypes of Δ*gerP* Mutants

**Figure 2:**
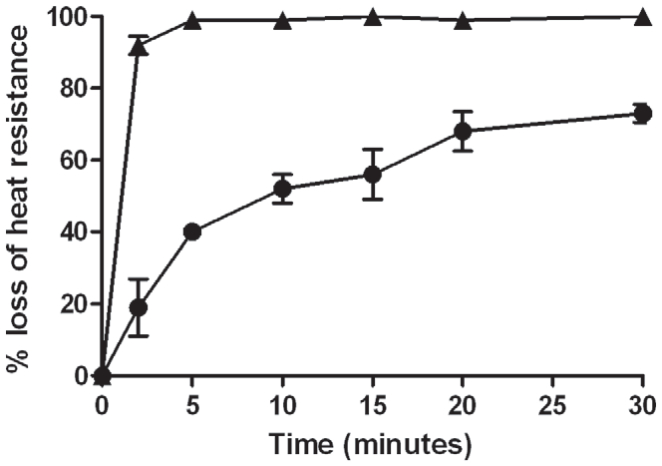
Germination phenotype of the Δ*gerP-null* mutant in response to L-alanine and inosine. Wild-type (▴) or Δ*gerP-null* (•) spore suspensions were incubated in 0.5 mM L-alanine and 1 mM inosine at 37°C for 0–30 minutes, and germination was measured as a loss in heat resistance. Data presented are mean values of 4 independent experiments with 2 different spore preparations. Error bars represent ± standard error of the mean.

In order to test the role of the GerP proteins during germination, markerless deletions were made in each of the six *gerP* genes, *gerPA-gerPF*, as well as a full operon deletion, denoted as Δ*gerP-null* (**Figure 1**). Two separate assays were performed to assess germination ability for each mutant in an L-alanine/inosine mixture. One assay measured the hallmark loss of heat resistance associated with the earliest moments of germination [31]. The other assay measured the decreased optical density (OD) associated with a germinating spore suspension [31]. Both assays are described in detail in the Methods section. Wild-type spores germinated rapidly, as measured by loss of heat resistance, reaching nearly full germination by 5 minutes (**Figure 2**). Δ*gerP-null* spores, however, exhibited a significant delay in germination rates, especially at early time points during the assay (**Figure 2**, **Table 2**). While wild-type spores had already reached 92% heat sensitivity at 2 minutes, only 19% of the Δ*gerP-null* spores were sensitive at the same time point. Over the course of the 30-minute assay, mutant spores germinated to a certain extent, but had still not reached 100%. Similar results were seen when germination was scored as a loss in OD_600_ (**Table 3**).

**Table 2:** Loss of heat resistance of *gerP* mutants in L-alanine and inosine.

	5 minutes^a^			30 minutes^a^		
Strain		*+ gerP*_n_ b	*+ gerP_ABCDEF_* c		*+ gerP_n_* b	*+ gerP_ABCDEF_* c
34F2	99%			100%		
Δ*gerPA*	41%	77%	83%	70%	86%	93%
Δ*gerPB*	36%	80%	80%	62%	91%	93%
Δ*gerPC*	36%	83%	85%	62%	94%	88%
Δ*gerPD*	36%	56%	81%	72%	88%	95%
Δ*gerPE*	48%	77%	79%	71%	95%	89%
Δ*gerPF*	84%	ND	ND	97%	ND	ND
Δ*gerPF_null_*	36%	88%	84%	73%	93%	92%
Δ*gerP-null*	40%		88	73%		91%

a Spores were incubated in 0.5 mM L-alanine and 1 mM inosine. Results are the average of four experiments with two independent spore preparations. Standard error of the mean was ≤8% of the mean in all instances.

b *gerP_n_* denotes a mutant complemented *in trans* with its respective *gerP* gene.

c *gerP_ABCDEF_* denotes a mutant complemented *in trans* with the full *gerP* operon.

**Table 3:** Decrease in optical density of *gerP* mutants in L-alanine and inosine.

	5 minutes^a^			30 minutes^a^		
Strain		*+ gerP_n_* b	*+ gerP_ABCDEF_* c		*+ gerP_n_* b	*+ gerP_ABCDEF_* c
34F2	38%			68%		
Δ*gerPA*	8%	30%	24%	18%	61%	59%
Δ*gerPB*	14%	32%	17%	45%	65%	55%
Δ*gerPC*	14%	36%	26%	52%	57%	56%
Δ*gerPD*	10%	23%	19%	45%	68%	58%
Δ*gerPE*	7%	32%	27%	28%	68%	58%
Δ*gerPF_null_*	10%	28%	24%	31%	62%	59%
Δ*gerP-null*	8%		32%	29%		62%

a Spores were incubated in 0.5 mM L-alanine and 1 mM inosine. ∼65% decrease in OD_600_ represents ∼100% germination. Results are the average of four experiments with two independent spore preparations. Standard error of the mean was ≤9% of the mean in all instances.

b *gerP_n_* denotes a mutant complemented *in trans* with its respective *gerP* gene.

c *gerP_ABCDEF_* denotes a mutant complemented *in trans* with the full *gerP* operon.

**Figure 3:**
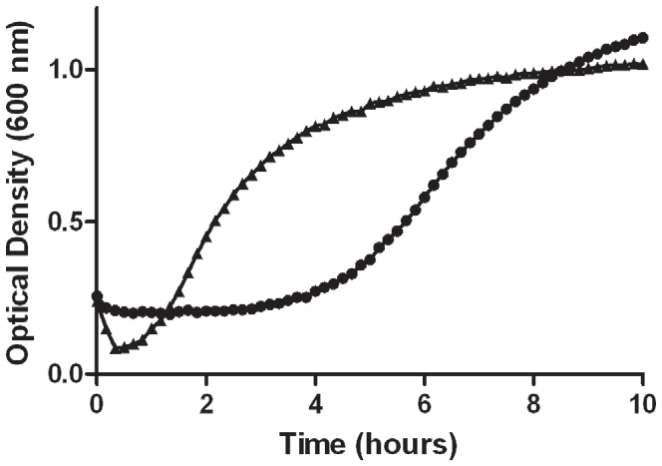
Outgrowth phenotype of the Δ*gerP-null* mutant in rich media. Wild-type (▴) or Δ*gerP-null* (•) spores in BHI. Spores were grown overnight at 37°C in a 96-well plate with shaking. Cultures were inoculated to a starting OD_600_ of 0.3. Data presented are representative of three independent experiments.

To better understand defects caused by deleting the *gerP* operon, growth curves were performed. Wild-type and mutant *B. anthracis* spores were tested for their ability to germinate, outgrow, and reach logarithmic growth in BHI medium. Spores were incubated in a 96-well plate with shaking overnight. Absorbance at OD_600_ was measured every 10 minutes in order to capture the characteristic loss in refractility during the first few minutes of germination [32]. In this germinant-rich environment, wild-type spores germinated and lost their refractility by 10 minutes, followed by outgrowth and exponential growth within 1 hour (**Figure 3**). Over the same period, Δ*gerP-null* spores exhibited no measurable decrease in optical density. They outgrew eventually and reached exponential growth, about 4 hours later than wild-type spores.

To further understand the role of the individual GerP proteins in germination, each of the individual *gerP* genes were removed, resulting in the strains Δ*gerPA*, Δ*gerPB*, Δ*gerPC*, Δ*gerPD*, Δ*gerPE*, and Δ*gerPF*. These mutants were also tested for the ability to germinate when exposed to L-alanine and inosine, as described above. All single deletion mutants exhibited a germination defect similar to the Δ*gerP-null* strain, with the exception of the Δ*gerPF* strain, which behaved much more like wild-type (**Tables 2** **and** **3**). Analysis of the *B. anthracis* genome sequence identified two putative *gerPF* orthologues (**Figure 1B and C**) [24]. These genes, GBAA 2341 (*gerPF2*) and GBAA 5052 (*gerPF3*, annotated as *gerPF-like*), had 79% and 59% nucleotide sequence identity and 95% and 52% amino acid similarity, respectively, to the *gerPF* gene located in the *gerP* operon. The two *gerPF* orthologues were deleted in the Δ*gerPF* background creating a triple *gerPF-*family mutant (Δ*gerPF_null_*). This triple mutant allowed us to better understand the role *gerPF* and its orthologues were playing during germination. Eliminating the contribution of all three *gerPF* orthologues resulted in a germination phenotype similar to those scored from other single *gerP* gene deletion mutants, suggesting that these three GerPF-family proteins may have overlapping functions (**Tables 2** **and** **3**).

Growth in BHI was also measured for the single gene deletion mutants Δ*gerPA*, Δ*gerPB*, Δ*gerPC*, Δ*gerPD*, Δ*gerPE*, and Δ*gerPF_null_*. Delays into logarithmic growth for these mutants, when compared to wild-type, ranged between 1 and 4 hours (data not shown). Though the severity of the growth delay varied from mutant to mutant, none of the single mutants exhibited the initial drop in OD_600_ characteristic of wild-type germinating spores. These data further support that each of the *gerP* genes is important for proper timing of spore germination and outgrowth.

Complementation of mutants was achieved *in trans* by placing a wild-type copy of the gene on plasmid pBKJ401, under control of the native *gerP* promoter (see Methods). The construct was then introduced into the mutant strain of interest. Complemented strains were tested for their ability to germinate in L-alanine and inosine. All of the mutants (Δ*gerPA*, Δ*gerPB*, Δ*gerPC*, Δ*gerPD*, Δ*gerPE*, Δ*gerPF_null_*, and Δ*gerP-null*) showed an increased ability to germinate when the deleted gene was supplied *in trans* (**Tables 2** **and** **3**). Additionally, a construct that carried the full length *gerP* operon was introduced into each mutant strain. Complementation with the full *gerP* operon was no better than complementation with the relevant individual gene (as measured by either germination assay; **Tables 2** **and** **3**), suggesting that the generation of these Δ*gerP* single mutants did not result in unintended polar effects. Collectively, these data suggest that each of the GerP proteins play a role in the process of germination, and that each appear essential to ensure rapid, wild-type level germination responses, as measured by these assays.

### Germination Behavior of Δ*gerP* Mutant Spores after Coat Removal

**Figure 4:**
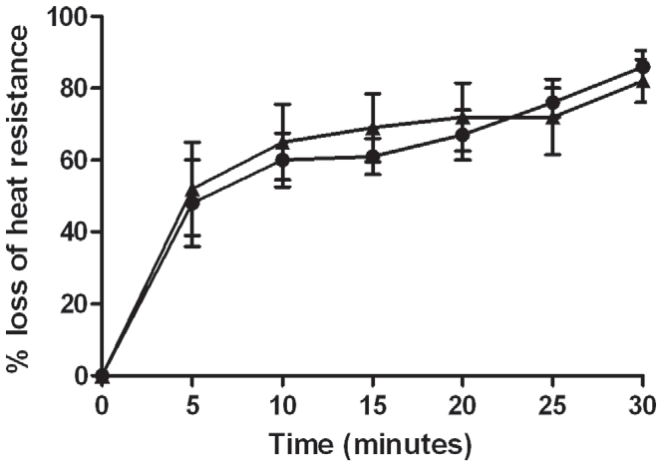
Germination behavior of spores after removal of spore coats. Wild-type (▴) or Δ*gerP-null* (•) spores were treated in UDS in order to remove the spore coat. Germination was then assessed in 0.5 mM L-alanine and 1 mM inosine at 37°C for 0–30 minutes. Data presented are mean values of 4 independent experiments with 2 different spore preparations. Error bars represent ± standard error of the mean.

As initially reported by Behravan *et al*., *B. cereus* GerP proteins were suggested be involved in spore coat structure and/or formation [23]. It is hypothesized that GerP proteins facilitate trans-coat transport of germinants and, therefore, that disruption of these proteins may impede this. Chemical removal of the selective spore coat layer in *gerP* mutant strains of *Bacillus subtilis* and *B. cereus* resulted in an increased ability of these spores to germinate [14], [23]. In our study, wild-type and mutant spores were chemically treated to remove their spore coats, and then tested for the ability to germinate in L-alanine/inosine. It should be noted that wild-type spores exhibited a reduced ability to germinate after the treatment to remove coats, suggesting that the treatment itself may inhibit a subpopulation from germinating (**Figure 4**). However, Δ*gerP-null* spores that were treated for coat removal germinated more rapidly than untreated Δ*gerP-null* spores, especially at the early time points of this assay. In fact, treated Δ*gerP-null* spores mimicked the germination profile of treated wild-type spores. The single deletion mutants in each of the first four genes of the operon, Δ*gerPA-gerPD*, also exhibited an increased ability to germinate after treatment, while Δ*gerPE* and Δ*gerPF_null_* did not show as much of an improvement (**Table 4**). These data suggest that at least some of the GerP proteins are important in spore coat permeability, and that their proper association with the coat is important for rapid spore germination.

**Table 4:** Loss of heat resistance of Δ*gerP* mutants after removal of spore coats.

Strain	2 minutes^a^	30 minutes^a^
34F2	52%	82%
Δ*gerPA*	56%	76%
Δ*gerPB*	48%	73%
Δ*gerPC*	48%	75%
Δ*gerPD*	47%	71%
Δ*gerPE*	40%	75%
Δ*gerPF_null_*	33%	82%

a Spores were incubated in 0.5 mM L-alanine and 1 mM inosine. Results are the average of four experiments with two independent spore preparations. Standard error of the mean was ≤11% of the mean in all instances.

### Germination in Ca-DPA

**Figure 5:**
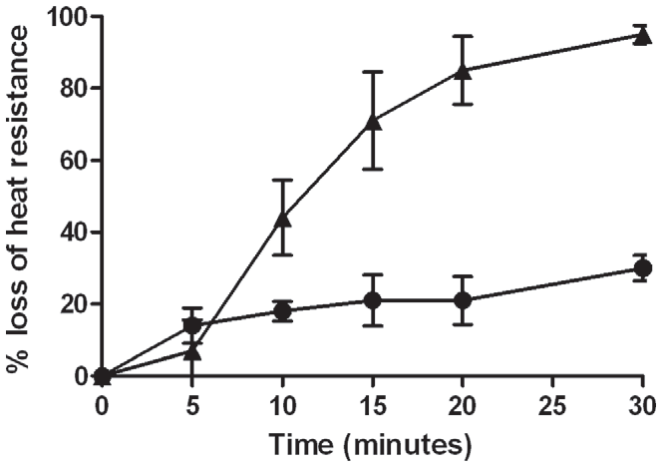
Germination phenotypes of of the Δ*gerP-null* mutant in response to Ca-DPA. Wild-type (▴) or Δ*gerP-null* (•) spore suspensions were incubated in 60 mM Ca-DPA at 37°C for 0–30 minutes, and germination was measured as a loss in heat resistance. Data presented are mean values of 4 independent experiments with 2 different spore preparations. Error bars represent ± standard error of the mean.

Exogenous Ca-DPA is able to bypass the need for germinant receptor stimulated germination of *B. subtilis* by directly activating the cortex lytic enzyme CwlJ. Active CwlJ then degrades the spore cortex, allowing full germination [20]. Wild-type *B. anthracis* spores readily germinated in the presence of Ca-DPA (**Figure 5**). The Δ*gerP-null* strain, however, exhibited a germination defect in this solution with only a 30% loss of heat resistant spores after 30 minutes (**Figure 5**). This defect was not just due to a slowed rate of germination, in contrast to the defect seen in amino acid germinants, as Δ*gerP-null* spores never fully germinated even hours after exposure to the Ca-DPA (data not shown). All of the mutants lacking a single *gerP* gene (or lacking the three *gerPF* family members) also exhibited this germination defect when compared to wild-type (**Table 5**). These data suggest that each of the *gerP* genes is required for Ca-DPA-mediated germination. They also suggest that, in addition to facilitating access of germinants to their receptors, the GerP proteins may be playing a different, and more essential, role in germination with exogenous Ca-DPA.

**Table 5:** Loss of heat resistance of Δ*gerP* mutants in Ca-DPA.

Strain	% heat sensitive^a^
Wild-type	95%
Δ*gerP null*	30%
Δ*gerPA*	19%
Δ*gerPB*	20%
Δ*gerPC*	20%
Δ*gerPD*	30%
Δ*gerPE*	35%
Δ*gerPF_null_*	44%

a Spores were incubated in 60 mM Ca-DPA for 30 minutes at 37°C. Results are the average of four experiments with two independent spore preparations. Standard error of the mean was ≤12% of the mean in all instances.

## Discussion

Upon entry into a host, the spores of *B. anthracis* must germinate in order to cause disease [15]. The ability of germinants to reach their receptors in the inner spore membrane is essential for germination, and yet the mechanism for this is still not well understood. The experiments described in this study have aimed to further our understanding of this process by characterizing the *B. anthracis* orthologue of the *gerP* operons from *B. subtilis* and *B. cereus* [23].

The GerP proteins in other *Bacillus* species have been shown to play an integral role in the process of germination. Strains that contain insertion mutations in the *gerPC* and *gerPE* genes of *B. subtilis* and *B. cereus* have severe germination defects when incubated in L-alanine or inosine. If the spore coats from these mutants are removed, the germination defect is alleviated [23]. It therefore appears that a role of GerP is to facilitate germinant access to receptors, or to contribute to spore coat permeability. We have extended the study of GerP to *B. anthracis*. Deletion of the full *gerP* operon resulted in a delay in germination, but was not as severe as what was reported for other *Bacillus* species. Additionally, the data from the growth experiments suggested that in *B. anthracis* the *gerP* operon is important, but not essential, for rapid germination and outgrowth. The Δ*gerP-null* mutant did not exhibit the characteristic decrease in optical density associated with germinating wild-type spores, likely because Δ*gerP* mutant spores germinated slowly, and in a non-synchronous manner. It is unlikely that this delay was simply due to a subpopulation of spores that were able to rapidly germinate, as wild-type and mutant spores had similar titers when plated on rich media (data not shown). These data suggest that the interaction of germinants with their receptors may be delayed in the absence of the GerP proteins in *B. anthracis*, but that once this occurs, germination proceeds normally. Additionally, the Δ*gerP-null* strain exhibited no attenuation compared to wild-type spores, when inoculated intratracheally in our DBA/2J mouse model (data not shown). Like the other *Bacillus* species examined, *B. anthracis* also showed increased germination after coat removal. Together these data suggest that the GerP proteins of *B. anthracis* are playing similar, but not identical roles, to the previously characterized *Bacillus* species, and that *B. anthracis* may possess another route of access for its nutrient germinants.

An additional phenotype not examined in other *Bacillus* species was the role GerP played in germination via exogenous Ca-DPA. This compound has been shown to directly activate the coat localized cortex lytic enzyme CwlJ in *B. subtilis*, alleviating the need for germinant receptors to initiate germination [20]. As the GerP proteins are likely located in the spore coat, we hypothesized that the GerP proteins might also provide Ca-DPA access to CwlJ. Indeed, the GerP proteins appeared to be essential for germination with exogenous Ca-DPA. In *B. subtilis*, endogenous Ca-DPA released from the spore's core during the initial moments of germination can also activate CwlJ [20]. It is possible that the GerP proteins are involved in this release of Ca-DPA from the spore's core. Additionally, when exogenous Ca-DPA was used as a germinant only a subpopulation of the mutant spores germinated, as no additional germination was seen over time. This suggests that a GerP independent route exists to allow in amino acid germinants, but Ca-DPA passage may be completely dependent on the GerP proteins.

For this study we also determined the contribution of each individual *gerP* gene to the germination phenotype. These analyses have not been performed for other *Bacillus* species. Five of the single-gene mutants had the same germination phenotype (Δ*gerPA-ΔgerPE*) as the Δ*gerP-null* strain, although only four exhibited rescued germination upon coat removal (Δ*gerPA-ΔgerPD*). The Δ*gerPF* germination phenotype, which closely resembled that of wild-type, was due to the presence of two *gerPF* orthologues on the chromosome. These additional copies of the *gerPF* gene may be playing additional, or complementary, roles in the germination pathway. One of these orthologues, *gerPF3* (GBAA5052) is predicted to be in an operon with the genes GBAA5053 and GBAA5054 [33]. Interestingly, gene GBAA5054 has a putative Excalibur calcium-binding domain and multiple S-layer homology (SLH) domains, which are typically anchored to the cell surface [34], [35]. Gene expression of GBAA5054 is upregulated during the early stages of sporulation, suggesting that it may be present in mature spores [36]. The presence of this calcium-binding protein in an operon with a *gerP*-like gene orthologue is intriguing, although no data exists to support the notion that it may play a role in Ca-DPA mediated germination. *B. subtilis* and *B. cereus* also have two *gerPF* orthologues located on their chromosomes, although their roles in germination have yet to be tested [25], [37]. As mentioned above, coat-removed Δ*gerPE* and Δ*gerPF_null_* spores did not exhibit increased germination, implying a role beyond the spore coat. They may play another role in germination, perhaps by providing a link between the receptors in the inner membrane and the remaining GerP proteins in the coat.

Collectively, the germination profiles of the individual Δ*gerP* mutants and the Δ*gerPF_null_* triple mutant suggest that each GerP protein is playing a crucial role in facilitating proper, rapid, germination. The GerP proteins play an important role in the early events of germination in all *Bacillus* species tested thus far. This role may involve allowing nutrients into the spore, Ca-DPA out of the spore, or both. More work remains in order to fully elucidate the role these proteins are playing, but that knowledge will lead us to a more complete understanding of how *B. anthracis* regulates the complex process of germination.
