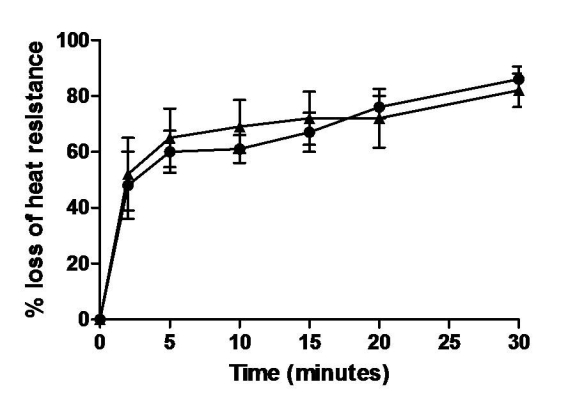# Correction: Role of the *gerP* Operon in Germination and Outgrowth of *Bacillus anthracis* Spores

**DOI:** 10.1371/annotation/8f1e2a19-cb97-4680-a6b9-40e0116e7842

**Published:** 2010-08-18

**Authors:** Katherine A. Carr, Brian K. Janes, Philip C. Hanna

Figure 4 was published with incorrectly labeled x-axis values. Please view the corrected figure here: 

**Figure:**